# No evidence to support a role for *Helicobacter pylori* infection and plasminogen binding protein in autoimmune pancreatitis and IgG4-related disease in a UK cohort

**DOI:** 10.1016/j.pan.2017.04.002

**Published:** 2017

**Authors:** Emma L. Culver, Wouter L. Smit, Caroline Evans, Ross Sadler, Tamsin Cargill, Mateusz Makuch, Lai-Mun Wang, Berne Ferry, Paul Klenerman, Eleanor Barnes

**Affiliations:** a Peter Medawar Building, Nuffield Department Medicine, Oxford University, UK; b Translational Gastroenterology Unit and NIHR Biomedical Research Centre, John Radcliffe Hospital, Oxford, UK; c Academic Medical Centre, Department of Gastroenterology and Hepatology, Amsterdam, The Netherlands; d Clinical Immunology Department, Churchill Hospital, Oxford, UK; e Department of Cellular Pathology, John Radcliffe Hospital, Oxford, UK

**Keywords:** Autoimmune pancreatitis, IgG4-related disease, IgG4, *Helicobacter pylori*, Adaptive immunity

## Abstract

**Background and objectives:**

*Helicobacter pylori* (*H.pylori*) plasminogen binding protein (PBP) has been proposed as an antigen triggering autoimmune pancreatitis (AIP), the pancreatic manifestation of IgG4-related disease (IgG4-RD). We investigated exposure to *H. pylori* infection, cytokine response and immunological memory to *H. pylori* PBP in a prospective IgG4-RD cohort in the UK.

**Methods:**

Clinical and endoscopic evidence of peptic ulceration, serological *H. pylori* exposure and serum IgG4 levels were obtained in 55 IgG4-RD patients and 52 disease controls (DC) with autoimmune or inflammatory conditions with an elevated serum IgG4. Gastric and duodenal tissues were assessed for *H. pylori* and immunostained for IgG4. B and T cell ELISpot and cytokine luminex assays were used to detect immune responses to *H. pylori* PBP.

**Results:**

85% of IgG4-RD patients had pancreatic and/or biliary disease, 89% had extra-pancreatic manifestations, and 84% had an increased serum IgG4. Clinical dyspepsia (35.2%), gastritis (58%), peptic ulceration (7.4%) and *H. pylori* colonisation (24%) in IgG4-RD was similar to DC. In IgG4-RD, gastric tissue contained a chronic inflammatory infiltrate with a low IgG4+ plasma-cell count (<10/HPF; range 1–4/HPF), and duodenal specimens had an increased IgG4 count (>10/HPF; range 7–54) compared with DC (p < 0.01). Th1 and Th2 cytokine response and immunological B-cell memory to *H. pylori* PBP did not differ between IgG4-RD and DC.

**Conclusions:**

In a prospective UK cohort, the prevalence of gastric ulceration, exposure to *H. pylori,* cytokine response and immunological memory to *H. pylori* PBP did not differ in IgG4-RD patients compared with DC. This study does not support a role for *H. pylori* PBP as a microbial antigen in IgG4-RD.

**Keywords for abstract:**

Peptic ulceration, Antigens, B cells, T cells, Interleukins, *Helicobacter pylori*.

## Introduction

Autoimmune pancreatitis (AIP) is the pancreatic manifestation of IgG4-related disease (IgG4-RD), a corticosteroid-responsive multi-system fibro-inflammatory condition of uncertain aetiology [1]. An increase in IgG4 B cells in the blood and tissue of patients, the presence of oligoclonal B cell and plasma cell populations, and human leukocyte antigen (HLA) class II associations, suggest a role for specific antigens in disease pathogenesis [2], [3], [4]. Over the last decade, *H. pylori*-related antigens have been implicated in the pathogenesis of AIP. One hypothesis states that AIP could be initiated when immune responses to specific infections generate cellular and humoral components that are cross-reactive to pancreatic tissue self-antigens in genetically predisposed individuals [5], [6]. This was based on reported associations between *H. pylori* and hepatobiliary autoimmune conditions (such as primary biliary cholangitis), as well as a high reported prevalence of peptic ulceration (35%) in AIP patients, a well-established consequence of gastric *H. pylori* infection [7], [8], [9], [10].

Early evidence of a relationship between *H. pylori*-related antigens and AIP came from *in silico* protein analysis, which identified structural homology between human carbonic anhydrase II (CA-II) and *H. pylori* α-carbonic anhydrase (α-CA) [11]. Elevated CA-II antibody levels were shown to correlate with serum IgG4 concentrations in AIP [12]. Subsequently, using a peptide library with purified immunoglobulin from AIP patients, Frulloni et al. identified *H. pylori* plasminogen-binding protein type A (PBP) as an antigen that was targeted by IgG antibodies in 95% of patients with AIP [13]. PBP shares sequence homology with the ubiquitin protein ligase E3 component n-recognin 2 (UBR2), an enzyme expressed in pancreatic acinar cells, to which most AIP patients also showed IgG reactivity [13]. Thus, a role for the microbial antigen PBP in the pathogenesis of AIP was postulated. To date there has been no validation of these findings.

In our study, we investigate the prevalence of gastric inflammation and ulceration, rates of *H. pylori* colonisation determined serologically and histologically, and assess evidence for *H. pylori* PBP as a microbial antigen in a prospective cohort of patients with AIP and IgG4-RD in the UK. Disease controls with inflammatory and autoimmune conditions were matched for comparison. We determine clinical history of dyspepsia and endoscopic evidence of gastritis and peptic ulceration in those who have undergone gastroscopy. Serum samples were tested for total IgG and IgG4 subclass, and gastric and duodenal specimens were immunostained for IgG4 antibodies. Serological evidence of *H. pylori*-specific IgG and histological evidence of *H. pylori* in gastric tissue was assessed. Finally, we sought evidence for *H. pylori*-specific immune responses using T-cell and B-cell ELISpot assays (using *H. pylori* PBP and lysate as antigens) and multiplex cytokine analysis.

## Methods

### Patient and control cohorts

The number of IgG4-RD patients that were assessed for study eligibility was 69. Inclusion criteria were adults over 18 years with a confirmed diagnosis of IgG4-RD meeting the diagnostic criteria defined below, and an *H. pylori* serology test taken at diagnosis. 55 patients with IgG4-RD referred to Oxford OUH NHS Trust from February 2005 to February 2013 and followed prospectively were included in the study (exclusions as no *H. pylori* serology tested (n = 16) or withdrew consent (n = 1)). Of these, 85% (47/55) had AIP and/or IgG4-related sclerosing cholangitis (IgG4-SC). An elevated serum IgG4 (>1.4  g/l) was detected in 84% (46/55) of these patients at presentation.

52 non-IgG4-RD patients with inflammatory and/or autoimmune conditions and an elevated serum IgG4 level, identified from March 2010 until February 2013 and followed prospectively were included as disease controls **(**Table S1**)**. Disease controls with an elevated serum IgG4 were chosen to minimise variability between IgG4-RD patients and the control population. Ethical approval for this study was provided by Oxfordshire Research Ethics Council (10/H0604/51). All patients provided written informed consent. The study was registered on the NIHR Portfolio UK Clinical Research Network.

### Diagnostic criteria for IgG4-RD

AIP and IgG4-SC were diagnosed using the Mayo HISORt (histology, imaging, serology, other organ involvement and response to corticosteroids) criteria from 2007 (AIP n = 36/36; IgG4-SC n = 33/33). AIP and IgG4-SC were diagnosed using the International Consensus Diagnostic Criteria (ICDC) for AIP type I from 2011 and Clinical Diagnostic Criteria (CDC) for IgG4-SC from 2012, and the criteria applied retrospectively to those enrolled prior to this (Definite AIP 28/36; Probable AIP n = 8/36; IgG4-SC 33/33) [14], [15], [16]. Patients with type II AIP (idiopathic duct-centric pancreatitis) were excluded as they represent a separate clinical entity [15]. Extra-pancreatic disease was diagnosed using the Comprehensive Diagnostic Criteria for systemic IgG4-RD (n = 49/55) [17]. The Boston Consensus Histopathological Criteria for IgG4-RD were applied to all patients with biopsy (n = 44) and resection (n = 17) specimens available [18]. Specific histological features included two of three morphological characteristics (lymphoplasmacytic infiltrate, storiform pattern of fibrosis, obliterative phlebitis) and an IgG4-positive plasma cell count of >10/HPF for biopsy and >50/HPF for resection specimens, with an IgG:IgG4 ratio of >40%. Non-diagnostic histology was available additionally in 20/55 patients.

### Clinical and endoscopic evidence of peptic ulceration and *H.pylori* infection

Patients were asked a routine set of questions in outpatient clinic that included a history of indigestion and/or dyspepsia, prior history of peptic ulceration, upper endoscopy and outcome, known *H. pylori* infection and previous eradication therapy. Details were confirmed over the telephone, by general practitioner and hospital records, and by retrieving endoscopy and histology reports.

### Serum IgG and IgG4 measurements

Blood samples were collected from both IgG4-RD patients and disease controls before steroids or other immunosuppressive medications were initiated. Total serum IgG and subclasses (IgG1 and IgG4) were measured by nephelometry (BNII analyser, Siemens, Surry, UK). An elevated serum IgG and IgG4 were defined as ≥16  g/l and ≥1.4  g/l respectively, the latter according to the Mayo HISORt criteria for AIP and local policy.

### Helicobacter on H&E gastric biopsies

Formalin fixed and paraffin embedded gastric biopsy samples from 17 IgG4-RD patients (all with AIP and/or IgG4-SC) and 16 non-IgG4-RD matched disease controls were assessed for evidence of *H. pylori* bacteria on routine H&E stain, where a minimum of 20 high-power fields (HPF) were examined (sensitivity 91% and specificity 100%). In areas of active gastritis, immunohistochemical staining for *H. pylori* bacteria was also performed (B-0471, dilution 1:200; DAKO, Carpinteria, CA) to increase detection rates (100% specificity and sensitivity combined) [19].

### Histological features and IgG4 immunostaining

Gastric and duodenal biopsy samples from the 17 patients with IgG4-RD and 16 disease controls were assessed for morphological features of IgG4-RD, namely a lymphoplasmacytic infiltrate, storiform fibrosis, obliterative phlebitis and the presence of eosinophils. Tissues were immunostained with monoclonal antibodies to human IgG and IgG4. An elevated IgG4 count, reported as the average number of IgG4-positive plasma cells in three HPF, was defined as >10/HPF per biopsy specimen. If IgG4 counts were elevated, an IgG4 to total IgG ratio was calculated and considered elevated when >40% in accordance with the Boston Histological Criteria for IgG4-RD [18].

### Immune responses to *H.pylori*

Three assays were used to detect *H Pylori-*specific immune responses including: (1) serology for the detection of antibodies; (2) T cell responses in Th1 (IFN-ϒ) and Th2 (IL-4) ELISpots and multiplex luminex Th2 cytokine assays; and (3) B cell ELISpot assays for immunological memory. The T and B cell assays used *H. pylori* PBP peptides and *H. pylori* lysate as antigens.

**(1) Helicobacter Serology** - All patients and disease controls were tested for serum *anti*-*H. pylori* IgG (Immulite 2000, Siemens) using a commercial kit validated at the John Radcliffe Hospital, Oxford. *Anti*-H. pylori IgG positivity suggests previous exposure to H. pylori infection, and does not determine current active infection.

**(2) T cell ELISpot -** Using the ELISpot assay (a technique for sensitive quantification of cytokine-secreting antigen specific T cells at the single-cell level) we determined the capacity of T cells stimulated with overlapping peptides derived from *H. pylori* PBP to produce INF-γ and IL4. A T cell ELISpot assay was performed with thawed peripheral blood mononuclear cells (PBMCs) rested overnight to detect Th1 (IFN-γ) and Th2 (IL4) responses, according to the manufacturer's instructions (Mabtech^®^). Using *H. pylori* PBP type A (strain 26695), a set of 111 linear and overlapping peptide epitopes (Mimotope^®^) was created, consisting of 15–18 amino acids with a 11 amino acid overlap **(**Table S2**)** [20]. The peptides were divided into 8 pools (300 μg/ml) and stored at −20 °C. *H.pylori* peptide lysate (Invitrogen^®^), containing the most common *H. pylori* strains, was used as a positive control for *H. pylori* exposure. Concanavalin A (ConA), a lectin extract that induces T cell proliferation and INF-γ release, was a positive control for cytokine release. A FEC peptide pool (mixed HLA class I restricted peptides from cytomegalovirus, Epstein Barr virus and influenza) was used to ensure quality control. In brief, 96-well ELISpot plates (Millipore^®^) were coated with 5 μg/ml IFN-γ (or IL4) monoclonal antibody solution (Mabtech^®^) for 15–24 h at 4 °C. The plates were washed with PBS and blocked with RPMI 1640 medium +10% FCS or 2 h at 37 °C. PBMCs were plated at 2 × 10^5^ cells/well in triplicate in the presence of pooled PBP peptides (3 μg/ml per well), and incubated for 15–24 h at 37 °C. Plates were washed 7 times with PBS/Tween 20 and incubated with 0.5 μg/ml biotinylated mouse anti-human IFN-γ (or IL4) antibody (Mabtech^®^) at room temperature (RT) for 2–4 h in the dark. After washing, an alkaline phosphatase-conjugated anti-biotin monoclonal antibody solution (Vector Laboratories^®^) was added and incubated at RT for 2 h in the dark. Plates were developed using 5-Bromo-4-chloro-3-indolyl phosphate with nitro blue tetrazolium chloride (BCIP/NBT) at RT until spots became macroscopically visible (10–15 min). Membranes were air-dried overnight. Image analysis was performed on a computer-assisted AID ELISpot reader designed to detect spots using predetermined criteria based on size, shape, and colorimetric density, and validated manually. The total response was calculated to all peptide pools after subtracting the mean background (DMSO). A positive response was defined as ≥ 50 spot-forming units (SFU) per million PBMCs and at least 3SD above background. Negative control values were <30 SFU.

**(3) Th2 Luminex cytokine assay -** Quantitative multiplexed immunoassays based on Luminex technology were performed using a Procarta immunoassay kit (polystyrene beads) from Affymetrix. Analytes tested were IL4, IL5, and IL13. Two 96-well filter plates were prepared by incubating with 150 μl of Reading buffer (containing sodium azide) for 5 min at RT. 50 μl of antibody polystyrene beads were added to each well, then washed once. Plasma samples from IgG4-RD patients, disease controls with elevated serum IgG4 and healthy controls with normal serum IgG4 level were tested.

**(4) B cell ELISpot assays** —B cell ELISpots were performed for the quantification of IgG-producing and IgG4-producing cells derived from the B memory cell compartment, based on a previously described assay [21]. Briefly, PBMCs were cultured for 6 days in the presence of a non-specific stimulants containing 83 ng/ml of pokeweed mitogen extract (PWM, Sigma-Aldrich^®^), 2.5 μg/ml of fully methylated CpG (invitrogen^®^) and a 1:5000 dilution of fixed *S. aureus*, Cowan (SAC, Calbiochem^®^), which stimulates polyclonal B memory cells to proliferate and differentiate into antibody-secreting cells (ASCs). After culture, supernatants were preserved at −80 °C for further cytokine analysis (Luminex). The PBMCs were recovered and plated out on a pre-coated plate with *H. pylori* PBP peptide pools, positive (*H.pylori* lysate) and negative controls (R10 medium), and IgG and IgG4 antibodies (Sanquin, 10 μg/ml). Alternatively, freshly isolated PBMCs were directly plated without stimulation to capture antibody-producing plasmablasts (ex vivo B cell ELISpot). Analysis was as per the T cell ELISpot assays.

### Statistical analysis

A Kruskal-Wallis test was used for multiple comparisons, Mann-Whitney for two group comparisons, and Chi squared or Fishers Exact for categorical variables, using Graphpad prism (v6.0). P values < 0.05 were considered to be statistically significant.

## Results

### Clinical characteristics

The majority of patients with IgG4-RD had pancreatic and/or biliary disease (85%; 47/55). Extra-pancreatic systemic manifestations were present in 89% (49/55), detected on cross-sectional imaging. An elevated serum IgG4 level at presentation was detected in 83.6% (46/55) (Table 1).

**Table 1:** Characteristics and *H.pylori* status of IgG4-RD patients and disease controls. The table shows the demographics and clinical characteristics in IgG4-RD, disease controls with an elevated serum IgG4. Abbreviations: M: male; F: female; HP: H.pylori; GU: gastric ulcer; DU: duodenal ulcer; UGIE: upper gastrointestinal endoscopy; HPF: high power field; +ve: positive; DC: disease controls. P values were calculated using Fisher's exact test; ns p ≥ 0.05,*p < 0.05,**p < 0.01,***p < 0.001,****p < 0.0001.

	IgG4-RD patients	Disease Controls	P value
Gender: Absolute M/F (% of male)	39/55 (71)	36/50 (72)	1.00 NS
Age, median (range) years	63.0 (30–82)	61 (19–89)	0.49 NS
High serum IgG4 (>1.4 g/l): Absolute (%)	46/55 (83.6)	42/50 (82.0)	1.00 NS
Autoimmune disease: Absolute (%)	15/55 (27.3)	35/50 (70.0)	<0.0001****
HP serology positive: Absolute (%)	13/55 (23.6)	16/50 (32.0)	0.386 NS
History of indigestion or dyspepsia: Absolute (%)	19/54 (35.2)	17/50 (34.0)	1.00 NS
Peptic ulcer: Absolute (%)	4/54 (7.4)	6/50 (12.0)	0.515 NS
Gastric Ulcer: Absolute (%)	1/4	2/6	N/A
Duodenal Ulcer: Absolute (%)	3/4	4/6	N/A
Upper Gastrointestinal Endoscopy: Absolute (%)	26/54 (48.1)	30/50 (60.0)	0.17 NS
Gastritis: Absolute (%)	15/26 (57.7)	16/30 (53.3)	0.793 NS
Duodenitis (*including 3 whipples specimens): Absolute (%)	5/29 (17.2)	1/30 (3.3)	0.103 NS
HP biopsy positive: Absolute (%)	4/17 (23.5)	2/16 (12.5)	0.656 NS
Duodenum IgG4 cells/HPF	7-54/HPF	0-5/HPF	<0.0001****
Stomach IgG4 cells/HPF	0-5/HPF	0-5/HPF	1.00 NS

### Indigestion and peptic ulceration

A history of dyspepsia was elicited in 35.2% (19/54) and peptic ulceration in 7.4% (4/54) of IgG4-RD patients. There was no difference in the prevalence of dyspepsia/indigestion (p = 1.0) or ulceration (p = 0.515) between patients with IgG4-RD and disease controls (Table 1).

### Upper endoscopy, biopsies and IgG4 immunostaining

Similar numbers of IgG4-RD patients (48%; 26/54) and disease controls (60%; 30/50) had an upper endoscopy with biopsies (p = 0.172). The main indications for endoscopy in IgG4-RD patients were to sample the duodenum/ampulla in patients with a head of pancreas mass or duodenal stricture (34.6%; 9/26), investigation of weight loss (19.2%; 5/26), or abdominal pain (19.2%; 5/26).

Biopsies of the gastric mucosa confirmed gastritis in 57.7% (15/26) of patients with IgG4-RD and 53.3% (16/30) of disease controls (p = 0.793). Helicobacter colonisation was identified in 23.5% (4/17) of patients with IgG4-RD and 12.5% (2/16) of disease controls (p = 0.656) (Table 1). All four IgG4-RD patients and one disease control with evidence of *H. pylori* colonisation received triple eradication therapy at the time of diagnosis.

Gastric tissue in patients with AIP and chronic active gastritis contained lymphocytes, neutrophils, macrophages and mast cells, without other histological hallmarks of IgG4-RD. Tissue IgG4 immunostaining performed in patients with *H. pylori*-associated gastritis did not show elevated gastric tissue IgG4 count (median 2 cells/HPF, range 1–4). *H. pylori* bacteria present in the chronic inflammatory infiltrate did not co-localise with IgG4-positive plasma cells (Fig. 6A and B). Interestingly, there was an increased IgG4-positive plasma cell count (>10/HPF) in 3 of 5 duodenal specimens of patients with AIP and duodenitis (Fig. 6C*).* This was not seen in disease controls with duodenitis, or in patients with IgG4-RD or disease controls without duodenal inflammation.

### *H.pylori* specific immune responses

**(1) *Anti*-*H. pylori* IgG serology:** 23.6% (13/55) of IgG4-RD patients and 30.7% (16/52) of disease controls were *anti-*H. pylori IgG seropositive (p > 0.05), suggesting prior H. pylori exposure (Table 1). Of these, 4/13 IgG4-RD patients and 1/16 disease controls had received H. pylori eradication therapy in the past.

**Fig. 1.**
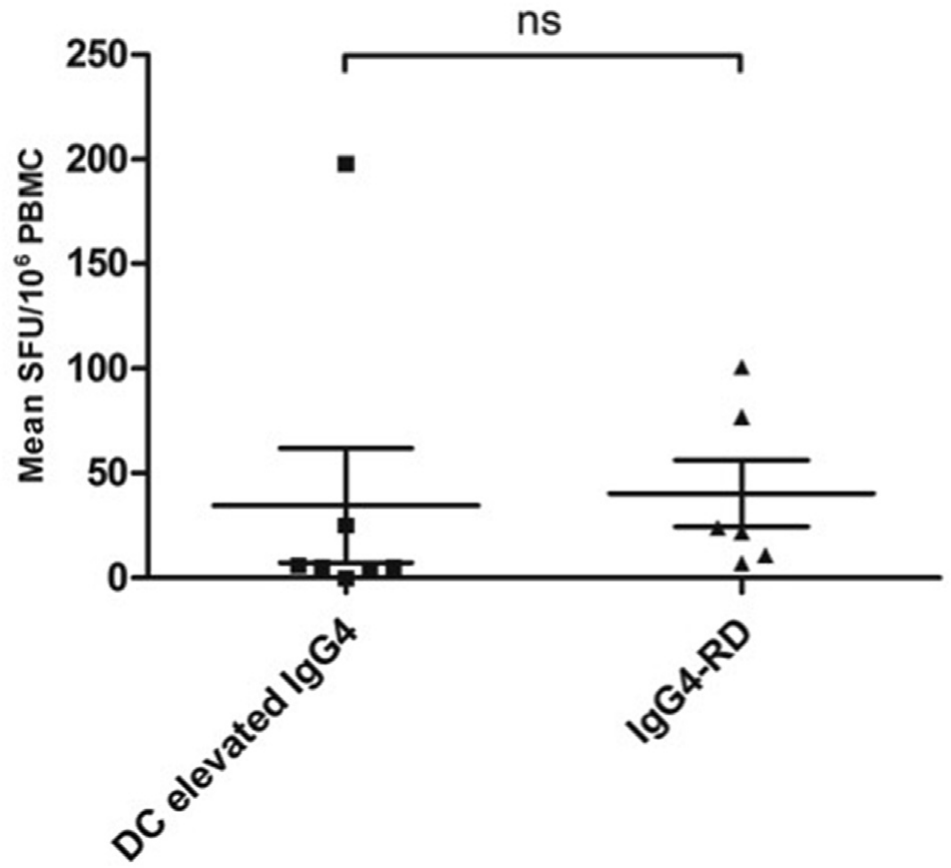
Total INF-γ T cell response to PBP in IgG4-RD patients and disease controls. Errors bars show the SEM. Mann-Whitney p values; ns ≥ 0.05. SFU: spot-forming units (per 10^6^ PBMCs).

**Fig. 2.**
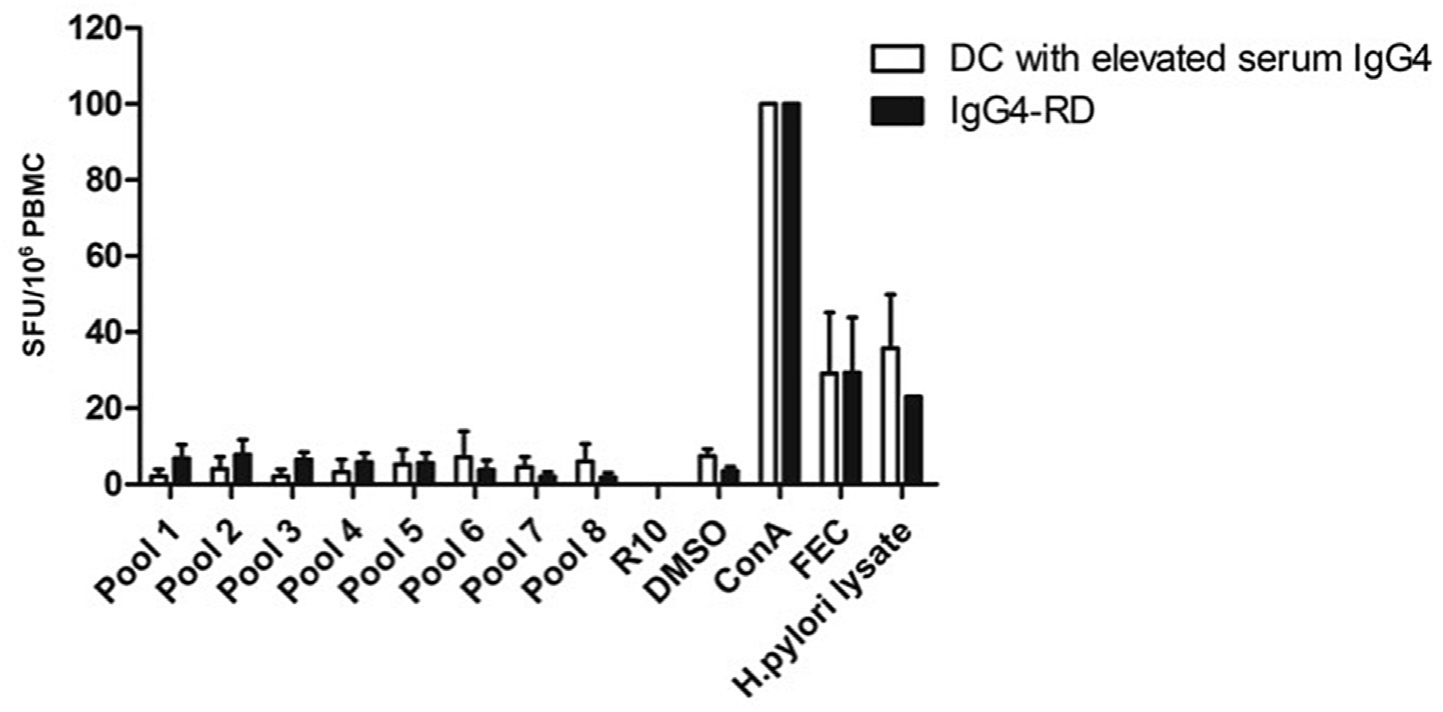
Mean IFN-γ T cell response to individual PBP peptide pools in IgG4-RD patients and disease controls. Peptide pools 1 to 8, positive controls (Con A, FEC and *H. pylori* lysate) and negative controls (R10-medium and DMSO) are shown on the X-axis. Con A: Concanavalin A; FEC: mixed HLA class I restricted peptides from cytomegalovirus, Epstein Barr virus and influenza; SFU: spot-forming units (per 10^6^ PBMCs).

**Fig. 3:**
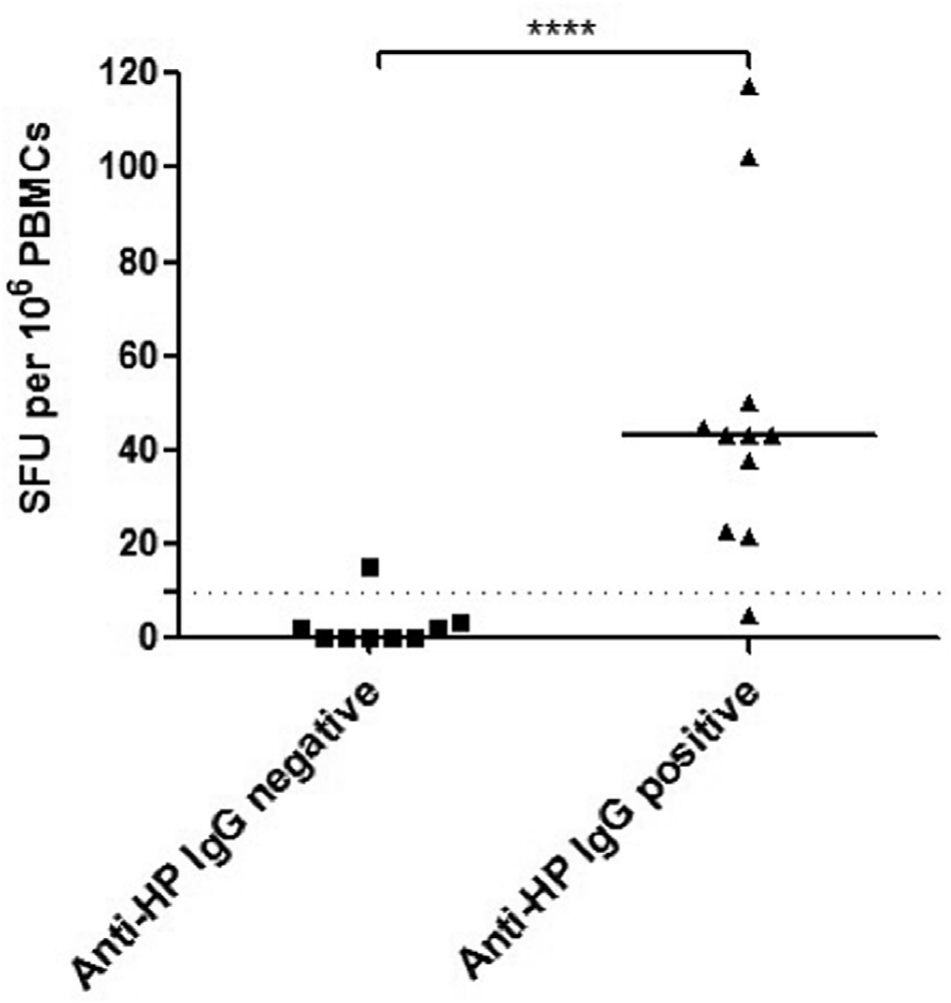
IFN-γ T cell response to *H. pylori* lysate in individuals with positive and negative *anti*-*H. pylori* IgG serology. Patients and disease controls were grouped and divided into those with positive and negative *anti*-*H. pylori* IgG serology. The dashed line represents the cut-off for a positive response: >10 spot-forming units (SFU) per 10^6^ PBMCs. Median values are given for both groups. Mann-Whitney p value; ****p < 0.0001.

**(2) T cell responses to *H. pylori* PBP using a T cell ELISpot:** We determined the capacity of T cells stimulated with overlapping peptides derived from *H. pylori* PBP to produce Th1 (INF-γ) and Th2 (IL4) responses. There was no difference in the total INF-γ T cell response to *H. pylori* PBP between patients with IgG4-RD and disease controls **(**Fig. 1**).** Similarly, there was no difference between INF-γ responses in the individual PBP peptide pools between patients with IgG4-RD and disease controls (Fig. 2***).*** T cell responses were further mapped down to the individual peptide level in one disease control with an increased T cell response (amino-acid sequence AKEERRY within pool 6), without evidence of *H. pylori*-specific PBP reactivity. A positive response to *H. pylori* lysate was demonstrated in patients with IgG4-RD and disease controls that were *anti*-*H. pylori* IgG seropositive, with a sensitivity of 90% and specificity of 89% **(**Fig. 3**).**

**Fig. 4:**
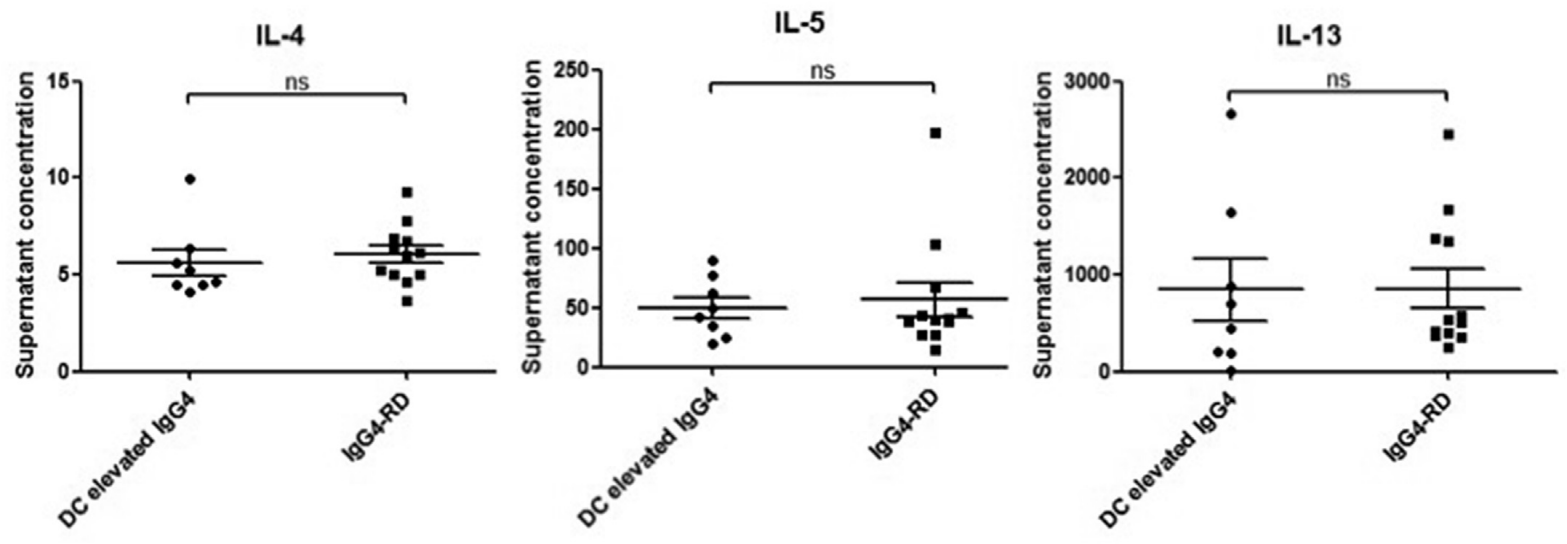
Th2 cytokine response in IgG4-RD patients, disease controls and healthy controls. Dot plots of the concentrations of the Th2 cytokines IL4, IL5 and IL13 in supernatants derived from cultured B cell ELISpots. In this assay PBMCs were stimulated non-specifically for 6 days, which elicits lymphocyte expansion and cytokine production. Kruskal-Wallis test with multiple comparisons p values; ns p ≥ 0.05.

**(3) T cell responses to *H. pylori* PBP using a Luminex multiplex cytokine assay:** We assessed Th2 cytokine responses (IL4, IL5 and IL13) in the supernatants of stimulated PBMCs after a 6-day culture. There were no differences in IL4, IL5 and IL13 concentrations between patients with IgG4-RD and disease controls (Fig. 4).

**Fig. 5:**
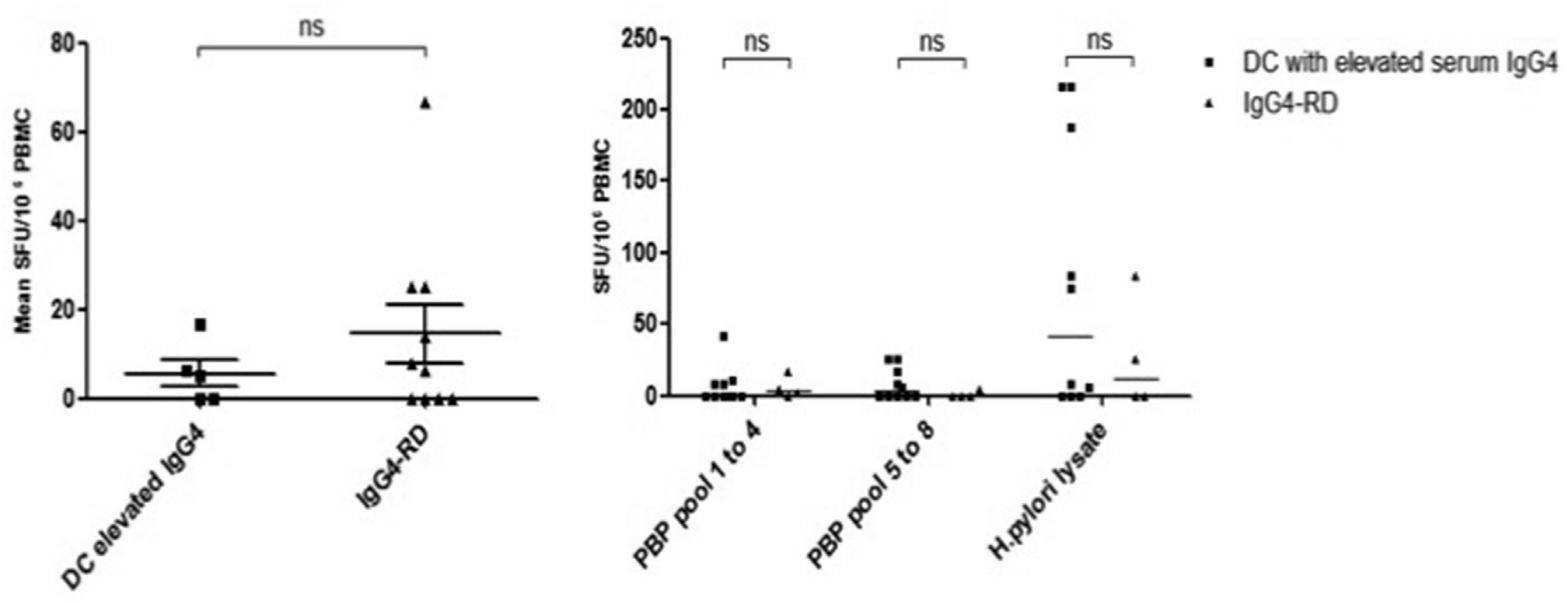
Total IgG responses in IgG4-RD patients and disease controls. **A (Left**): Total IgG response in IgG4-RD patients and disease controls with elevated serum IgG4. Errors bars show the SEM. Mann-Whitney p values; ns p ≥ 0.05. **B (Right)**: IgG response to PBP peptide pools 1–4 and 5–8, and *H. pylori* whole lysate in IgG4-RD patients and disease controls with an elevated serum IgG4. Errors bars show the median. SFU: spot-forming units (per 10^6^ PBMCs).

**(4) IgG and IgG4 B cell memory responses to PBP**: To determine if patients with IgG4-RD exposed to *H. pylori* infection have a memory B cell-mediated antibody response to PBP peptide, we performed a cultured B cell ELISpot assay to detect antigen-specific B memory cells that were non-specifically stimulated to differentiate into antibody-producing plasma cells. There was no difference between the total IgG response in patients with IgG4-RD and disease controls **(**Fig. 5A**).** There was no increased IgG or IgG4 response to PBP peptide pools (Fig. 5B**).** An increased IgG response was demonstrated in *anti*-*H. pylori* IgG positive compared to negative groups, suggesting increased immunogenicity to *H. pylori* in those previously exposed.

**Fig. 6:**
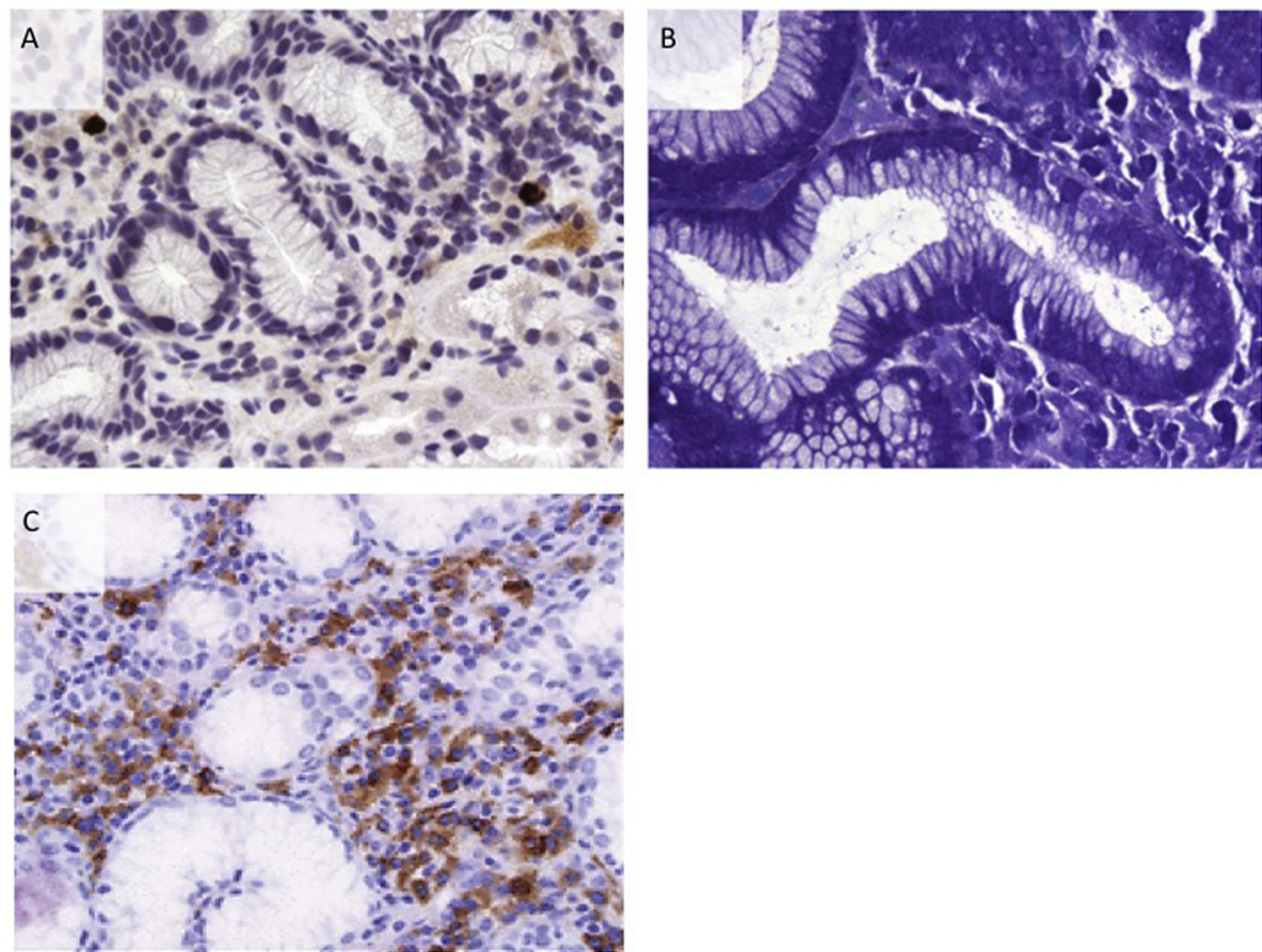
**(A):** DAB-positive (brown) IgG4-positive plasma cells in gastric tissue of an AIP patient. (**B):***H. pylori*-associated gastritis with lymphocytes and neutrophils in an AIP patient. (**C):** DAB-positive (brown) IgG4-positive plasma cells in a duodenal biopsy in a patient with AIP (IgG4 cells >10 per high power field).

## Discussion

Studies have implicated *H. pylori* in the pathogenesis of AIP through mechanisms of molecular mimicry. We sought to investigate exposure to *H. pylori* and the presence of immune response to *H. pylori* PBP in a prospective cohort of patients with type I AIP and IgG4-RD. We found no evidence to support increased exposure to *H. pylori* in patients with IgG4-RD compared with disease controls with an elevated serum IgG4 or age-matched population statistics in the UK. There was also no evidence to support immunological memory to *H. pylori* PBP in patients with IgG4-RD compared with disease controls. Based on our data, there is no evidence to support *H. pylori* PBP as a microbial antigen in patients with type I AIP or IgG4-RD.

Seroprevalence of *anti*-*H. pylori* IgG in our UK IgG4-RD cohort was 23.6%, which is comparable to Chinese AIP cohorts (27.5%), but considerably lower than the Italian AIP cohort (84%) from which *H. pylori* PBP reactivity was originally identified [13], [22]. This may be explained by an over-representation of type II AIP in the Italian cohort given that the percentage of patients with elevated serum IgG4 in this group was low (50%) compared to UK and Asian cohorts (82–95%). These type II AIP patients were excluded from our study. UK population statistics indicate a similar *H. pylori* seroprevalence of 25% in age-matched individuals [23], whereas our disease controls with elevated serum IgG4 had a seroprevalence of 31%, in line with reported associations between *H. pylori* and autoimmune conditions [8], [9].

In a susceptible host, *H. pylori* infection determines chronic active gastritis, gastric and duodenal ulceration, gastric cancer and MALT-lymphoma [24]. Clinical history and endoscopic evidence of peptic ulceration was described in only 7.2% of patients with IgG4-RD, much lower compared to the 22.5 and 34.8% of gastric ulcers at endoscopy described in Chinese and Japanese AIP patients, respectively [10], [22]. These discrepancies may originate from regional differences in *H. pylori* colonisation and circulating strains causing ulceration, ethnicity-related susceptibility factors to both AIP and *H. pylori* infection, or that 17% of our IgG4-RD cohort did not have pancreatic and/or biliary disease [25]. The ulceration seen in our cohort was predominantly duodenal. The single gastric ulcer was found in the antrum and was non-linear, which was different to the distribution and morphology described at endoscopy in one AIP series [19]. *H. pylori* -associated gastritis was detected in 23.5% of patients with IgG4-RD, similar to the 20% described in a Chinese AIP cohort [22]. This *H. pylori*–associated chronic active gastritis was characterised by an infiltration of the gastric epithelium and lamina propria by B and T cells, plasma cells, neutrophils, macrophages and mast cells. There were a few gastric IgG4-positive plasma cells (<10/HPF) that did not co-localise with *H. pylori* bacteria. However, in those AIP patients with duodenitis, there were abundant duodenal IgG4-positive plasma cells (>10/HPF), which may be important in providing support for diagnosis. One other study reported high IgG4/IgG ratios in the gastric mucosa of AIP patients with concomitant gastritis, although *H. pylori* bacteria were not identified [26].

Proposed theories regarding the initiation of the immune response in IgG4-RD suggest triggering by auto-antigens that may activate antigen-presenting cells in genetically predisposed individuals. Frulloni et al. showed that a majority of AIP patients were IgG-positive to both *H. pylori* PBP and human UBR2, proteins that share sequence homology. Potential cross reactivity of serum from AIP patients was also shown ex vivo [13]. In these patients one would thus expect the presence of immunological memory to these peptides. To assess this in our cohort, we used multiple immune assays to detect *H. pylori* specific B and T cell responses. Despite evidence of immunogenicity to *H. pylori* peptides and lysate in patients and controls that were *anti*-*H. pylori* IgG positive reflecting prior exposure, assessment of T cell cytokine response and B cell immunological memory did not reveal reactivity against *H. pylori* PBP. This is supported by a recent study, where sera from *H. pylori* –positive controls did not show higher reactivity towards the PBP peptide than *H. pylori* –negative sera, using ELISA with PBP rabbit polyclonal antibodies [27]. Furthermore, in a study using nested PCR to detect conserved *H. pylori* DNA in pancreatic tissue and juice from AIP and chronic pancreatitis patients, there was no evidence of its presence, supporting the concept that direct infection by *H. pylori* in pathogenesis is unlikely [28].

Differences in findings between our study and Frulloni *et al* may be explained by variations in AIP patient characteristics (demographics, comorbidities and serum IgG4 levels), phylogeographic differentiation between pathogenic *H. pylori* strains that express altered forms of PBP on the surface, and genetic susceptibility that is partly determined by HLA expression, which differs between ethnic regions [29], [30]. Furthermore, it is conceivable that differences in methodology between studies could play a role. For instance, the use of different means to detect antigen (T and B cell ELISpot rather than DELFIA assay) or the use of linear overlapping peptide sequences rather than whole protein that may have resulted in suboptimal epitope presentation or insufficient binding due to the loss of the three-dimensional conformation. However, we believe our approach is sufficiently sensitive to detect immunogenic exposure to *H. Pylori*, and would therefore be detected if this patient cohort if this organism was to play a significant role in disease pathogenesis. Alternative immune mechanisms that might implicate *H. pylori* involvement in AIP, but not be detected by our assays, such as epitope spreading, non-specific bystander activation or the presence of different *H. pylori* antigens (e.g. *H. pylori* carbonic anhydrase) [11] may be considered, although the fact that there was no evidence of increased exposure to *H. Pylori* bacteria in our IgG4-RD cohort make these unlikely.

In summary, our data shows that exposure to *H. pylori* in patients with type I AIP and IgG4-RD is much lower than previously reported, and similar to both autoimmune and inflammatory conditions with an elevated serum IgG4 level and matched population statistics. Whether *H. Pylori* and PBP is involved in type 2 AIP is a question that remains be addressed. However, the data does not support a role for *H. pylori* PBP as the initiating antigen in the pathogenesis of type 1 AIP or systemic IgG4-RD.

## Author contributions

EC, EB, and PK were involved in the study concept and design. EC and WS obtained funding, collected, analysed and interpreted data, performed statistical analysis, and drafted the original manuscript. CS and WS supervised B-cell ELISpots. RS processed samples for serological measurement and supervised B-cell ELISpots and Luminex analysis. MM analysed and interpreted ELISpot and Luminex data. TC collected patient samples and validated clinical data. LMW was responsible for immunostaining of histological specimens and their interpretation. BF is the head of the clinical immunology department and responsible serological processing and interpretation. EB is the principal investigator on the IgG4-RD NIHR Portfolio study and EB and PK co-supervised the project. All authors critically reviewed and approved the final manuscript for intellectual content.

## Appendix A Supplementary data

SupplementaryTable S1 Disease characteristics of the non-IgG4-RD disease controls with elevated serum IgG4 levels. SupplementaryTable S1

SupplementaryTable S2 Sequence of *H.pylori* plasminogen binding peptide type A (strain 26695), and 111 linear and overlapping peptide epitopes, consisting of 15–18 amino acids with an 11 amino acid overlap. SupplementaryTable S2
