# Supplementary material for: No evidence to support a role for Helicobacter pylori infection and plasminogen binding protein in autoimmune pancreatitis and IgG4-related disease in a UK cohort
Source: Pancreatology. 2017 May-Jun;17(3):395–402. doi: 10.1016/j.pan.2017.04.002 (PMC5459459; doi:10.1016/j.pan.2017.04.002)
Supplement: SupplementaryTable S1 — Disease characteristics of the non-IgG4-RD disease controls with elevated serum IgG4 levels. [file mmc1.docx]

| **Autoimmune and or inflammatory condition (Disease Controls)** | **Number of patients** |
| --- | --- |
| Primary sclerosing cholangitis  (Co-existing Inflammatory bowel disease) | 27  (20) |
| Chronic Pancreatitis | 5 |
| Gallbladder disease with cholestasis | 4 |
| Rheumatoid arthritis | 3 |
| Cryptogenic cirrhosis | 3 |
| Biliary stricture | 2 |
| Large vessel vasculitis | 2 |
| Coeliac disease | 2 |
| Interstitial lung disease and bronchiectasis | 2 |
| Sjögrens syndrome | 1 |
| Hypereosinophilic syndrome | 1 |
