# Supplementary material for: No evidence to support a role for Helicobacter pylori infection and plasminogen binding protein in autoimmune pancreatitis and IgG4-related disease in a UK cohort
Source: Pancreatology. 2017 May-Jun;17(3):395–402. doi: 10.1016/j.pan.2017.04.002 (PMC5459459; doi:10.1016/j.pan.2017.04.002)
Supplement: SupplementaryTable S2 — Sequence of H.pylori plasminogen binding peptide type A (strain 26695), and 111 linear and overlapping peptide epitopes, consisting of 15–18 amino acids with an 11 amino acid overlap. [file mmc2.docx]

| ***H.pylori* PBP Type A amino acid sequence** |
| --- |
| MLRLLIGLLLMSFISLQSASWQEPLRVSIEFVDLPKKIIRFPAHDLQVGEFGFVVTKLSDYEIVNSEVVIIAVENGVATAKFRAFESMKQRHLPTPRMVARKGDLVYFRQFNNQAFLIAPNDELYEQIRATNTDINFISSDLLVTFLNGFDPKIANLRKACNVYSVGVIYIVTTNTLNILSCESFEILEKRELDTSGVTKTSTPFFSRVEGIDAGTLGKLFSGSQSKNYFAYYDALVKKEKRKEVRIKKREEKIDSREIKREIKQEAIKEPKKANQGTQNAPTLEEKNYQKAERKLDAKEERRYLRDERKKAKATKKAMEFEEREKEHDERDEQETEGRRKALEMDKGDKKEERVKPKENEREIKQEAIKEPSDGNNATQQGEKQNAPKENNAQKEENKPNSKEEKRRLKEEKKKAKAEQRAREFEQRAREHQERDEKELEERRKALEAGKK |

| **Peptide No** | **Sequence** |
| --- | --- |
| 1 | MLRLLIGLLLMSFIS |
| 2 | LIGLLLMSFISLQSA |
| 3 | LLMSFISLQSASWQE |
| 4 | FISLQSASWQEPLRV |
| 5 | QSASWQEPLRVSIEF |
| 6 | WQEPLRVSIEFVDLP |
| 7 | LRVSIEFVDLPKKII |
| 8 | IEFVDLPKKIIRFPA |
| 9 | DLPKKIIRFPAHDLQ |
| 10 | KIIRFPAHDLQVGEF |
| 11 | FPAHDLQVGEFGFVV |
| 12 | DLQVGEFGFVVTKLS |
| 13 | GEFGFVVTKLSDYEI |
| 14 | FVVTKLSDYEIVNSE |
| 15 | KLSDYEIVNSEVVII |
| 16 | YEIVNSEVVIIAVEN |
| 17 | NSEVVIIAVENGVAT |
| 18 | VIIAVENGVATAKFR |
| 19 | VENGVATAKFRAFES |
| 20 | VATAKFRAFESMKQR |
| 21 | KFRAFESMKQRHLPT |
| 22 | FESMKQRHLPTPRMV |
| 23 | KQRHLPTPRMVARKG |
| 24 | LPTPRMVARKGDLVY |
| 25 | RMVARKGDLVYFRQF |
| 26 | RKGDLVYFRQFNNQA |
| 27 | LVYFRQFNNQAFLIA |
| 28 | RQFNNQAFLIAPNDE |
| 29 | NQAFLIAPNDELYEQ |
| 30 | LIAPNDELYEQIRAT |
| 31 | NDELYEQIRATNTDI |
| 32 | YEQIRATNTDINFIS |
| 33 | RATNTDINFISSDLL |
| 34 | TDINFISSDLLVTFL |
| 35 | FISSDLLVTFLNGFD |
| 36 | DLLVTFLNGFDPKIA |
| 37 | TFLNGFDPKIANLRK |
| 38 | GFDPKIANLRKACNV |
| 39 | KIANLRKACNVYSVG |
| 40 | LRKACNVYSVGVIYI |
| 41 | CNVYSVGVIYIVTTN |
| 42 | SVGVIYIVTTNTLNI |
| 43 | IYIVTTNTLNILSCE |
| 44 | TTNTLNILSCESFEI |
| 45 | LNILSCESFEILEKR |
| 46 | SCESFEILEKRELDT |
| 47 | FEILEKRELDTSGVT |
| 48 | EKRELDTSGVTKTST |
| 49 | LDTSGVTKTSTPFFS |
| 50 | GVTKTSTPFFSRVEG |
| 51 | TSTPFFSRVEGIDAG |
| 52 | FFSRVEGIDAGTLGK |
| 53 | VEGIDAGTLGKLFSG |
| 54 | DAGTLGKLFSGSQSK |
| 55 | LGKLFSGSQSKNYFA |
| 56 | FSGSQSKNYFAYYDA |
| 57 | QSKNYFAYYDALVKK |
| 58 | YFAYYDALVKKEKRK |
| 59 | YDALVKKEKRKEVRI |
| 60 | VKKEKRKEVRIKKRE |
| 61 | KRKEVRIKKREEKID |
| 62 | VRIKKREEKIDSREI |
| 63 | KREEKIDSREIKREI |
| 64 | KIDSREIKREIKQEA |
| 65 | REIKREIKQEAIKEP |
| 66 | REIKQEAIKEPKKAN |
| 67 | QEAIKEPKKANQGTQ |
| 68 | KEPKKANQGTQNAPT |
| 69 | KANQGTQNAPTLEEK |
| 70 | GTQNAPTLEEKNYQK |
| 71 | APTLEEKNYQKAERK |
| 72 | EEKNYQKAERKLDAK |
| 73 | YQKAERKLDAKEERR |
| 74 | ERKLDAKEERRYLRD |
| 75 | DAKEERRYLRDERKK |
| 76 | ERRYLRDERKKAKAT |
| 77 | LRDERKKAKATKKAM |
| 78 | RKKAKATKKAMEFEE |
| 79 | KATKKAMEFEEREKE |
| 80 | KAMEFEEREKEHDER |
| 81 | FEEREKEHDERDEQE |
| 82 | EKEHDERDEQETEGR |
| 83 | DERDEQETEGRRKAL |
| 84 | EQETEGRRKALEMDK |
| 85 | EGRRKALEMDKGDKK |
| 86 | KALEMDKGDKKEERV |
| 87 | MDKGDKKEERVKPKE |
| 88 | DKKEERVKPKENERE |
| 89 | ERVKPKENEREIKQE |
| 90 | PKENEREIKQEAIKE |
| 91 | EREIKQEAIKEPSDG |
| 92 | KQEAIKEPSDGNNAT |
| 93 | IKEPSDGNNATQQGE |
| 94 | SDGNNATQQGEKQNA |
| 95 | NATQQGEKQNAPKEN |
| 96 | QGEKQNAPKENNAQK |
| 97 | QNAPKENNAQKEENK |
| 98 | KENNAQKEENKPNSK |
| 99 | AQKEENKPNSKEEKR |
| 100 | ENKPNSKEEKRRLKE |
| 101 | NSKEEKRRLKEEKKK |
| 102 | EKRRLKEEKKKAKAE |
| 103 | LKEEKKKAKAEQRAR |
| 104 | KKKAKAEQRAREFEQ |
| 105 | KAEQRAREFEQRARE |
| 106 | RAREFEQRAREHQER |
| 107 | FEQRAREHQERDEKE |
| 108 | AREHQERDEKELEER |
| 109 | QERDEKELEERRKAL |
| 110 | EKELEERRKALEAGK |
| 111 | KELEERRKALEAGKK |
